# Glucocorticoids Alter Bone Microvascular Barrier via MAPK/Connexin43 Mechanisms

**DOI:** 10.1002/adhm.202404302

**Published:** 2025-01-20

**Authors:** Eun‐Jin Lee, Peter Lialios, Micaila Curtis, James Williams, Yoontae Kim, Paul Salipante, Steven Hudson, Mandy B. Esch, Moshe Levi, Joanna Kitlinska, Stella Alimperti

**Affiliations:** ^1^ Department of Biochemistry and Molecular and Cellular Biology School of Medicine Georgetown University Washington DC 20057 USA; ^2^ Biological and Biomedical Engineering Center School of Medicine Georgetown University Washington DC 20057 USA; ^3^ Microsystems and Nanotechnology Division Physical Measurement Laboratory National Institute of Standards and Technology Gaithersburg MD 20899 USA; ^4^ Department of Chemistry and Biochemistry College of Computer Mathematical and Natural Sciences University of Maryland College Park MD 20742 USA; ^5^ Materials Science and Engineering Division Material Measurement Laboratory National Institute of Standards and Technology Gaithersburg MD 20899 USA

**Keywords:** 3D bicellular platform, bone, Connexin43, glucocorticoids, MAPK, microfluidics, microvasculature, osteoporosis

## Abstract

Glucocorticoids (GCs) are standard‐of‐care treatments for inflammatory and immune disorders, and their long‐term use increases the risk of osteoporosis. Although GCs decrease bone functionality, their role in bone microvasculature is incompletely understood. Herein, the study investigates the mechanisms of bone microvascular barrier function via osteoblast‐endothelial interactions in response to GCs. The animal data shows that prednisolone (Psl) downregulated the osteoblast function and microvessel number and size. To investigate the role of GCs in bone endothelial barrier function further, a bicellular microfluidic in vitro system is developed and utilized, which consists of three‐dimensional (3D) perfusable microvascular structures embedded in collagen I/osteoblast matrix. Interestingly, it is demonstrated that GCs significantly inhibit osteogenesis and microvascular barrier function by interfering with endothelial‐osteoblast interactions. This effect is triggered by MAPK‐induced phosphorylation of connexin43 (Cx43) at Ser282. Collectively, this study sheds light on microvascular function in bone disorders, as osteoporosis, and permits to capture dynamic changes in endothelial‐bone interactions under GCs by dissecting the MAPK/Cx43 mechanism and proposing this as a potential target for bone diseases.

## Graphical abstract

Osteoporosis induced by Glucocorticoids (GCs) is a common complication of long‐term GC use in patients with inflammatory and autoimmune conditions, often leading to an increased fracture risk. A 3D bicellular endo‐osteo microfluidic platform is developed to explore pathogenic mechanisms underlying GCs‐induced osteoporosis, highlighting a novel MAPK/Connexin43 (Cx43) pathway that regulates bone microvascular barrier function and osteoblast‐endothelial interactions.

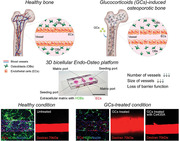

## 1 Introduction

Autoimmune diseases, including rheumatoid arthritis, inflammatory bowel disease, systemic lupus erythematosus, and gout,^[^ ^1^, ^2^, ^3^ ^]^ are often debilitating and, in some cases, life‐threatening. Glucocorticoids (GCs), such as prednisone, methylprednisolone, and dexamethasone, are potent treatments for them.^[^ ^4^, ^5^, ^6^ ^]^ Despite their beneficial clinical effects, patients under prolonged GC therapy are at high risk of developing bone‐related diseases, such as osteoporosis.^[^ ^7^, ^8^, ^9^ ^]^ GCs‐induced osteoporosis is the most prevalent type of secondary osteoporosis and the most common cause before 50 years of age, which has been linked with a higher incidence of fracture and bone loss.^[^ ^7^, ^10^ ^]^

The predominant effect of GCs on bone is linked to the loss of function of osteoblasts.^[^ ^11^, ^12^ ^]^ GCs at high doses significantly reduce the numbers of osteoblasts and osteocytes, resulting in decreased bone formation.^[^ ^13^, ^14^ ^]^ In addition, they affect the osteoclast‐osteoblast balance by increasing osteoclast activity via upregulation of receptor activator of NF‐κB ligand (RANKL) and decrease of osteoprotegerin, which is secreted by osteoblasts.^[^ ^15^ ^]^ Also, they increase renal calcium excretion and decrease gastrointestinal calcium absorption, resulting in reduced serum calcium, which increases the secretion of parathyroid hormone (PTH) and subsequently increases bone resorption.^[^ ^16^, ^17^ ^]^ Overall, the loss of the activity of osteoblasts and osteoclasts and the imbalance in their intracellular communication disrupt bone tissue homeostasis and lead to the development of osteoporosis.

Interestingly, osteoporosis, as in many pathogenic conditions in bone, is associated with alterations in bone microvasculature.^[^ ^18^ ^]^ In general, bone microvasculature plays a significant role in bone function and regeneration. It actively participates in the cartilaginous callus formation, periosteal response, bony callus, and bone formation and remodeling.^[^ ^19^, ^20^ ^]^ The mechanisms involved in these events require the intracellular communication of endothelial cells with mesenchymal stromal cells, osteoblast precursor cells, macrophages, pericytes, and endothelial progenitor cells via secretion of osteogenic and angiogenic growth factors, such as vascular endothelial growth factor (VEGF) and platelet‐derived growth factor.^[^ ^21^, ^22^, ^23^, ^24^ ^]^ During these steps, improper neo vessel formation and loss of microvascular function led to the development of bone diseases.^[^ ^25^, ^26^, ^27^, ^28^ ^]^ Consequently, osteoporosis has been accompanied by a decrease in the number of sinusoidal capillaries and blood flow.^[^ ^29^, ^30^, ^31^ ^]^

There is little to no in‐depth, quantitative knowledge regarding the impact of GCs on bone microvasculature and endothelial‐bone intracellular communication. Herein, we have successfully developed a three‐dimensional (3D) biomimetic microvascular bone platform composed of a perfusable blood vessel surrounded by osteoblasts. This 3D system allows us to investigate bicellular mechanisms involved in GCs‐induced osteoporosis under biologically relevant conditions than conventional two‐dimensional (2D) in vitro models and to avoid the use of animal models, which are laborious and not suitable for detailed mechanistic studies in a spatiotemporal manner. Using this model, we have shown that GCs decrease the osteogenic capacity of bone cells and the barrier function of endothelial cells. In addition, we have found that in the presence of GCs, bicellular interactions of endothelial cell‐osteoblasts are mediated via MAPK/connexin43 (Cx43) signaling. Collectively, this study sheds light on microvascular function in osteoporosis and permits us to capture dynamic changes in endothelial cell‐bone interactions under GCs by dissecting the Cx43 mechanism and proposing this as a potential target for bone diseases.

## 2 Results

### 2.1 GCs Decrease Osteogenic and Endothelial Function In Vivo

**Figure 1:**
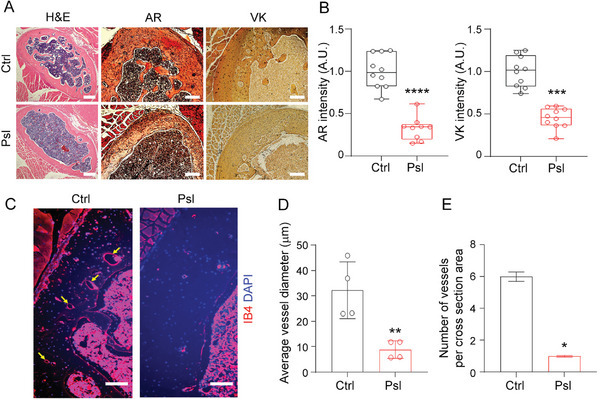
Glucocorticoids decrease osteogenic and endothelial function in vivo. A) Hematoxylin and eosin (H&E), Alizarin red (AR), and Von kossa (VK) staining of hind limb sections from mouse implanted with prednisolone (Psl) pellets for 60 days. Mouse hind limbs were dissected, fixed, and decalcified, and the cross sections with trabecular region were stained with H&E, AR (red), or VK (dark brown). Representative images are shown. Scale bars, 500 µm (H&E) and 200 µm (AR & VK). B) Quantitative analysis of the AR‐positive or VK‐positive surface intensity in bone tissue sections. n = 10 mice. C) Representative image of Isolectin‐4 (IB4) staining of bone sections. Yellow arrows indicate IB4 positive (red). Scale bars, 100 µm. D) Graph showing the blood vessel diameter in control and Psl‐treated bone tissues. N = 3, n = 4. E) Number of blood vessels per cross‐section area for control and Psl‐treated bone tissues. The quantitative data are expressed as means ± SD. N = 3, n = 4; significant differences: *, p‐value < 0.05, **, p‐value < 0.01, ***p‐value < 0.001, ****p‐value < 0.0001.

GCs have been responsible for the changes in bone function and have been involved in the development of osteoporosis.^[^ ^7^ ^]^ Our in vivo results showed that prednisolone (Psl)‐treated mice demonstrated lower osteogenic function. Specifically, bone tissues from Psl‐treated animals showed ≈3 times lower mineralization based on calcium deposition (measured by Alizarin red (AR) staining) and mineral deposition (measured by Von Kossa (VK) staining) (**Figure** **1**A,B; Figure S1, Supporting Information). In addition, we evaluated the size and number of microvessels in the Psl‐treated tissues, which were stained with Isolectin‐4 (IB4). Our results showed that Psl‐treated bone tissues had ≈ 3 times smaller vessel diameter and a lower number of blood vessels, indicating that Psl affects both osteogenesis and microvasculature in bone (Figure 1C–E).

### 2.2 GCs Decrease Osteogenesis

We aimed to extend the in vivo studies by evaluating the role of GCs in osteoblasts and endothelial cells. Initially, our study tested the role of hydrocortisone (Hyd), prednisone (Pred), and Psl in osteogenic function. After 3 weeks under osteogenic differentiation conditions (osteoblast mineralization medium, MM), Human osteoblasts (HOBs) demonstrated an increase in alkaline phosphatase (ALP) by ≈11 times, AR by ≈4.5 times, and VK by ≈6.4 times compared to untreated conditions (osteoblast growth medium, GM) (Figure S2, Supporting Information). To expand our studies and evaluate the effect of GCs in osteogenesis, HOBs cultured in MM were treated with 200 nmol L^−1^ Hyd, 100 nmol L^−1^ Pred, and 100 nmol L^−1^ Psl for 1 week to 4 weeks. Specifically, ALP, AR, and VK assays demonstrated significant downregulation of osteogenesis under those conditions compared to untreated cells cultured in MM conditions (Ctrl) (Figure S3, Supporting Information). In addition, the osteoimage mineralization assay showed a decrease in hydroxyapatite formation (HA) (Figure S4, Supporting Information). Taken together, our results demonstrated that GCs negatively affect the osteoblast function by downregulating osteogenesis.

### 2.3 Manufacturing a 3D Vascularized Bone Platform

**Figure 2:**
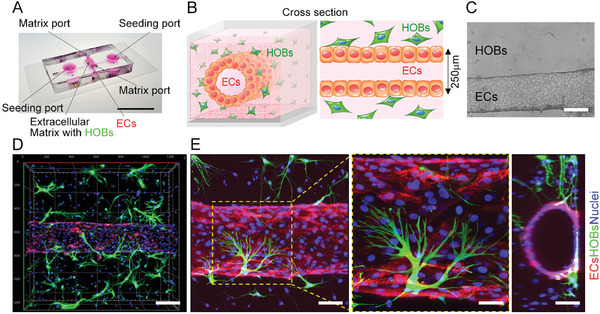
Engineering three‐dimensional (3D) bicellular Endo‐Osteo platform. A) Photograph of 3D microfluidic device using 3D printing scaffold. Scale bar, 1 cm. B) Schematic showing the cross‐sectional area of the 3D platform. The channel of endothelial cells (ECs) is formed in a 3D extracellular matrix containing human osteoblasts (HOBs) within a microfabricated PDMS gasket. C) Representative image of engineered microvessel in a 3D extracellular matrix with HOBs under phase contrast microscopy. Scale bar, 50 µm. D,E) Representative confocal immunofluorescence images capturing the formed 3D perfusable endothelial vessel (ECs; red) surrounded by HOBs (green) and nuclei for DAPI (blue). Scale bars, 200 µm (1st picture), 100 µm (2nd & 4th pictures), and 50 µm (3rd picture).

Next, we aimed to study the role of GCs in bone microvascular function. To explore this, we engineered and utilized a novel 3D bicellular microfluidic model (**Figure** **2**A,B). The 3D model recapitulates the structure and function of microvasculature in bone by integrating two major cell types, named HOBs and Human endothelial cells (ECs) (Figure 2C). Specifically, the system was comprised of a 3D cylindrical perfusable blood vessel channel, consisting of ECs embedded within a collagen I/fibrinogen/fibronectin matrix containing HOBs (Figure 2D,E; Figure S5, Supporting Information). Finally, the microvascular barrier function was assessed by real‐time microscopy, capturing 70 kDa dextran diffusion, as shown in Figure S6 (Supporting Information), and in our previous studies.^[^ ^32^, ^33^ ^]^

### 2.4 Mimicking GCs‐Induced Osteoporosis in the 3D Vascularized Bone Platform

**Figure 3:**
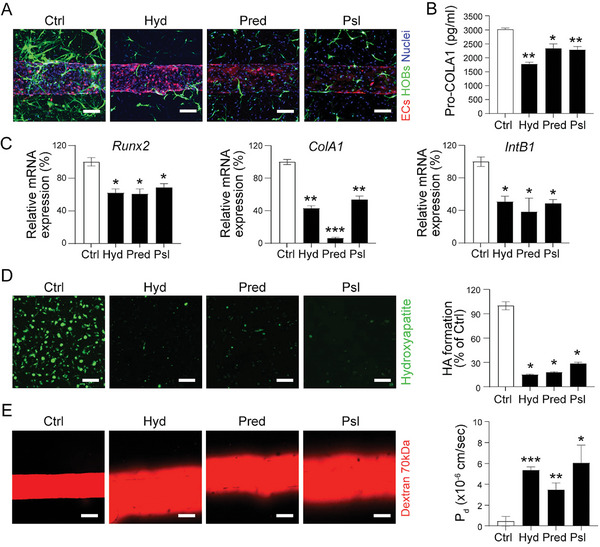
Glucocorticoids downregulate osteoblast function and disrupt the endothelial barrier. A) Representative confocal immune‐fluorescence images for 18 days mineralized samples capturing the formed endothelial vessel (ECs; red) surrounding by HOBs (green) in the presence of GCs such as hydrocortisone (Hyd), prednisone (Pred), and prednisolone (Psl). B) Culture supernatants were collected, and collagen type 1 protein levels (Pro‐COLA1) were analyzed by ELISA. N = 3. C) RNA was isolated from the devices cultured in the presence of Hyd, Pred, and Psl. The expression of osteoblast‐specific genes runt‐related transcription factor 2 (Runx2), collagen type I alpha 1 (COLA1), and Integrin beta‐1 (IntB1) was examined by RT‐qPCR. The transcript levels were normalized to 18S. N = 3. D) The 3D matrix in the presence of Hyd, Pred, and Psl were stained for hydroxyapatite (HA) formation (green). Scale bars, 200 µm. Right panel, Quantitative analysis of hydroxyapatite (HA) formation. N = 3. E) Representative endothelial integrity images on the devices using fluorescent‐labeled 70 kDa Texas red dextran. Scale bars, 200 µm. Graph demonstrates the endothelial leakiness in 18 days mineralized samples by diffusive permeability coefficient (*P_d_*) in the presence of GCs. The quantitative data are expressed as means ± SD. At least N = 3, n = 6; significant differences: *, p‐value < 0.05, **, p‐value < 0.01, ***, p‐value < 0.001.

As a next step, we examined the effect of GCs on bone microvascular barrier function. The 3D microfluidic systems were treated with 200 nmol L^−1^ Hyd, 100 nmol L^−1^ Pred, and 100 nmol L^−1^ Psl for 18 days (**Figure** **3**A). GCs downregulated the secretion of Pro‐Collagen I alpha 1 (Pro‐COLA1) from HOBs by ≈1.7 times (Hyd), ≈ 1.3 times (Pred), and ≈ 1.3 times (Psl) compared to Ctrl in MM (Figure 3B). RT‐qPCR analysis showed a decrease of osteogenic markers, runt‐related transcription factor 2 (Runx2), collagen type I alpha 1 (COLA1), and Integrin beta‐1 (IntB1) in the presence of GCs (Figure 3C). In addition, the osteoimage mineralization assay showed a decrease in hydroxyapatite formation (HA) by ≈ 9.8 times (Hyd), ≈ 7.5 times (Pred), and ≈ 3 times (Psl) (Figure 3D). Finally, the endothelial barrier function was measured by the extravasation of fluorescently labeled 70 kDa dextran from the lumen. After 18 days, microvascular leakiness was increased by ≈ 3.5 times (Hyd), ≈ 2.3 times (Pred), and ≈ 3.4 times (Psl) compared to Ctrl (Figure 3E), indicating that GCs downregulate osteogenesis and endothelial barrier function.

### 2.5 GCs regulate Microvascular Barrier Function via Cx43

**Figure 4:**
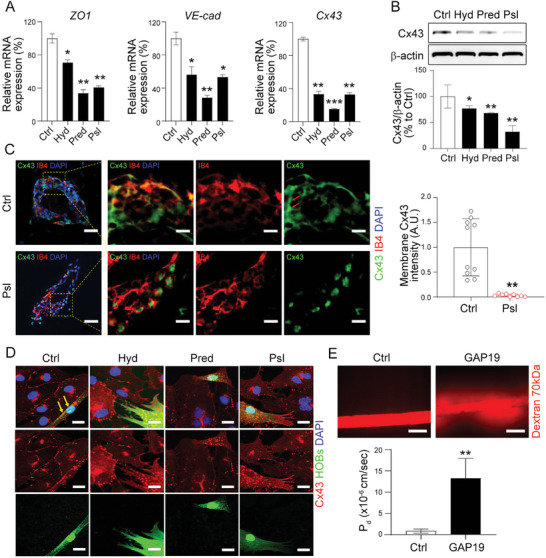
Glucocorticoids control endothelial and osteoblasts interactions via Cx43. A) RNA was isolated from 3D devices in the presence of Hyd, Pred, and Psl after 18 days. The expression of cell‐cell adhesion genes including zonula occludens‐1 (ZO‐1), vascular endothelial cadherin (VE‐cad), and connexin43 (Cx43) was examined by RT‐qPCR. The transcript levels were normalized to 18S. N = 3. B) The protein expression of Cx43 in the presence of GCs was analyzed by immunoblotting. The below panel shows the quantification of Cx43 levels compared to control. N = 5. C) Representative images of immunofluorescent signals for Cx43 (green), IB4 (red), and DAPI (blue) in bone sections from a mouse implanted with Psl pellets for 60 days or corresponding placebo control. Scale bars, 20 µm and 5 µm (zoom in images); Red arrows indicate the presence of Cx43 in the cell membrane area. Quantitative analysis of the Cx43‐positive membrane intensity in sections from each mouse stain. n = 10. D) Immunostaining for Cx43 (red) for co‐culture system of ECs and EGFP‐labeled HOBs (green). The nuclei were stained with DAPI (blue). Membrane localization of Cx43 is indicated by yellow arrows. Scale bars, 20 µm. E) Representative endothelial integrity images on the devices treated with GAP19 (5 µm; 30 min) using fluorescence labeled 70 kDa dextran. Scale bars, 100 µm. The below graph demonstrates the endothelial leakiness by diffusive permeability coefficient (*P_d_*) for control and GAP19 treated. The quantitative data are expressed as means ± SD. N = 3, n = 6; significant differences: *, p‐value < 0.05, **, p‐value < 0.01, ***, p‐value < 0.001.

To explore how GCs alter bone endothelial barrier function, we investigated whether GCs alter key cell‐cell adhesion molecules, such as zonula occludens‐1 (ZO‐1), vascular endothelial cadherin (VE‐cad), and Cx43, which have been implicated into the regulation of barrier function.^[^ ^32^ ^]^ Specifically, RT‐qPCR co‐culture analysis demonstrated that the transcription levels of ZO‐1, VE‐cad, and Cx43 have been downregulated in the presence of GCs for 18 days (**Figure** **4**A). Interestingly, Cx43, which is expressed in HOBs and ECs,^[^ ^34^ ^]^ demonstrated a ≈ 1.3 times (Hyd), ≈ 1.5 times (Pred), and ≈ 3 times (Psl) decrease compared to untreated conditions (Ctrl) in co‐culture conditions (Figure 4B). Finally, bone microvessels from Psl‐treated animals demonstrated ≈ 80% lower membrane Cx43 expression compared to untreated mice (Figure 4C). Therefore, functional perturbation in Cx43 expression is an attractive candidate for dissecting the mechanisms involved in bone microvascular function. Interestingly, confocal immunofluorescent staining demonstrated that even short exposure (30 min) of EC‐HOBs in GCs led to the loss of Cx43 from the membrane area (junctional sites) (Figure 4D), indicating that GCs controlled these heterotypic EC‐HOBs interactions via Cx43. Finally, utilizing the 3D bicellular microfluidic system, we showed that blockage of Cx43 via GAP19 inhibitor (5 µM) increased leakiness by ≈ 9.5 times, indicating that Cx43 is essential for the regulation of microvascular barrier function (Figure 4E).

### 2.6 GCs Regulate Microvascular Barrier Function via the MAPK/Cx43 Pathway

**Figure 5:**
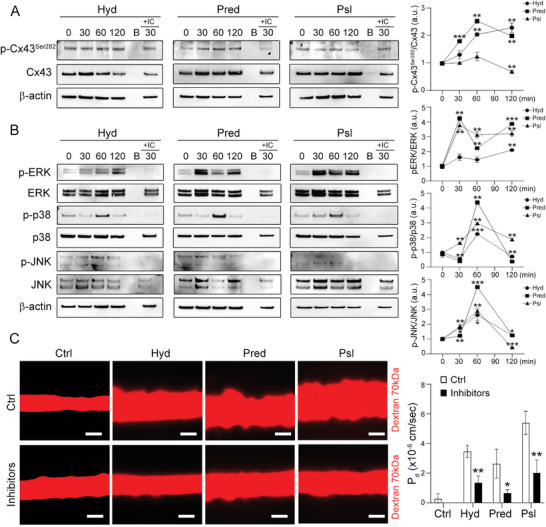
Glucocorticoids regulate microvascular barrier function via MAPK/Cx43. A) Left, Western blot analysis showing GCs‐induced activation of Phospho‐Cx43^Ser282^, and Cx43 in co‐cultures of HOBs and ECs following stimulation with Hyd, Pred, and Psl for 30 min, 60 min, and 120 min. Inhibitor treatment was applied for 30 min. Right, Quantitative analysis of the Western blot results. N = 3. B) Left, Western blot analysis showing GCs‐induced activation of MAPKs (ERK, p38, and JNK) in co‐cultures of HOBs and ECs following stimulation with Hyd, Pred, and Psl for 30 min, 60 min, and 120 min. Inhibitor treatment was applied for 30 min. Right, Quantitative analysis of the Western blot results. N = 3. C) Representative images showing endothelial integrity in control and inhibitor‐treated group exposed to GCs on the devices for 30 min, using fluorescence‐labeled 70 kDa Texas Red dextran. Scale bar, 100 µm. Right panel, Graph demonstrates the endothelial leakiness measured by the diffusive permeability coefficient (*P_d_*) for control and inhibitor‐treated groups with GCs. The quantitative data are expressed as means ± SD. N = 3, n = 3; significant differences: *, p‐value < 0.05, **, p‐value < 0.01, ***, p‐value < 0.001.

**Figure 6:**
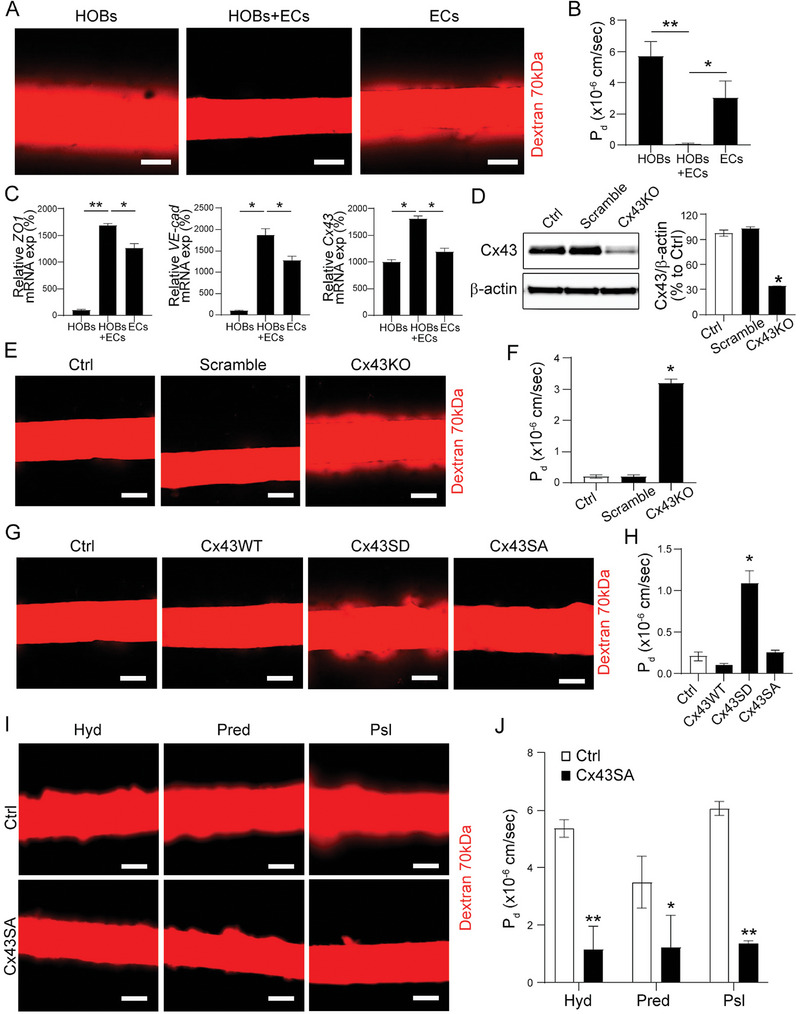
Human osteoblast cells enhance endothelial barrier function via Cx43. A) Representative images showing endothelial integrity on the devices, using fluorescence‐labeled 70 kDa dextran. Scale bar, 100 µm. B) Graph demonstrates the endothelial leakiness measured by diffusive permeability coefficient (*P_d_*) for only HOBs, HOBs+ECs, and ECs. At least N = 3. C) RNA was isolated in HOBs, HOBs+ECs, and only ECs from 3D matrix devices. The expression of cell‐cell adhesion genes such as ZO‐1, VE‐cad, and Cx43 was examined by RT‐qPCR. The transcript levels were normalized to 18S. N = 3. D) The protein expression of Cx43 was determined by immunoblotting from genetically engineered HOBs; Control (Ctrl), Scramble CRISPR (Scramble), and Cx43 CRISPR (Cx43KO). β‐actin was used as a loading control. The right panel shows the quantification of Cx43 levels compared to Ctrl. N = 3. E) Representative images showing endothelial integrity of Ctrl, Scramble, and Cx43KO on the devices, using fluorescence labeled 70 kDa Texas red dextran. Scale bar, 100 µm. F) Graph demonstrates the endothelial leakiness measured by diffusive permeability coefficient (*P_d_*) for Ctrl, Scramble, and Cx43KO. N = 3. G) Representative images showing endothelial integrity of genetically engineered HOBs; Cx43 wild‐type (Cx43WT), phospho‐mimetic Cx43^Ser282^, Cx43SD, and blocking phospho‐mimetic Cx43^Ser282^, Cx43SA on the devices using fluorescence labeled 70 kDa Texas red dextran. Scale bar, 100 µm. H) Graph demonstrates the endothelial leakiness measured by diffusive permeability coefficient (*P_d_*) in Ctrl, Cx43WT, Cx43SD, and Cx43SA. N = 3, n = 6. I) Representative images showing barrier function in the devices seeded with ECs and genetically engineered HOBs expressing Cx43SA treated with Hyd, Pred, and Psl, using fluorescence labeled 70 kDa Texas red dextran. Scale bar, 100 µm. J) Graph demonstrates the endothelial leakiness measured by diffusive permeability coefficient (*P_d_*) in Cx43SA with Hyd, Pred, and Psl. The quantitative data are expressed as means ± SD. N = 3, n = 6; significant differences: *, p‐value < 0.05, **, p‐value < 0.01.

Next, we investigated molecular pathways that are involved in the regulation of Cx43 in EC‐HOBs co‐culture conditions. Specifically, our results showed that the presence of GCs had no effect on the Cx43 expression compared to untreated conditions (Ctrl) in short‐term exposure (30 min, 60 min, and 120 min). Also, we demonstrated that in GCs conditions, there is an increase in the phosphorylated isoforms of Cx43 at Ser282 (p‐Cx43^Ser 282^) (**Figure** **5**A), which are phosphorylated via the MAPK pathway.^[^ ^35^, ^36^, ^37^, ^38^ ^]^ To this end, our results showed that GCs increased the phosphorylation levels of MAPK/ERK pathway molecules, ERK 1/2, p38, and JNK. Next, in the presence of GCs, the cells were treated with an inhibitor cocktail for the MAPK pathway, consisting of 25 µmol L^−1^ SP600125 (JNK inhibitor), 10 µmol L^−1^ SCH772984 (ERK1/2 inhibitor), 30 µmol L^−1^ Losmapimod (p‐p38 inhibitor). Our results demonstrated significant downregulation in p‐Cx43^Ser 282^ (Figure 6A) and p‐ERK1/2, p‐38, and p‐JNK (Figure 5B). To further investigate the role of MAPK/Cx43 in regulating bone microvascular barrier function, a dextran 70 kDa assay was performed by utilizing the 3D bicellular microfluidic system. Our results showed that inhibition of the MAPK pathway decreased microvascular leakiness induced by GCs by ≈ 3 times (Hyd), ≈ 4 times (Pred), and ≈ 3 times (Psl), indicating that the MAPK pathway controls barrier function via regulation of Cx43 (Figure 5C).

### 2.7 Endothelial Barrier Function is Regulated by Cx43‐Mediated Heterotypic HOB‐ECs Interactions

Next, we utilized this 3D bicellular microfluidic system to identify targets responsible for the changes in bone microvascular barrier function and, ultimately, bone pathophysiology. Our study focuses on the bicellular interactions of endothelial cells and osteoblasts. We evaluated the role of HOBs in bone microvascular barrier under normal and GC treatment conditions. We found that the presence of HOBs reduced leakage of 70 kDa dextran into the interstitial area compared to microvessels containing ECs alone by ≈18 times (**Figure** **6**A,B). Under those conditions, RT‐qPCR analysis showed that endothelial markers of ZO‐1 and VE‐cad were upregulated by ≈ 32% and ≈ 47%, respectively, indicating that HOBs enhance barrier function and junctional molecule expression (Figure 6C). Interestingly, Cx43 significantly increased (≈ 51%) under the co‐culture conditions. Therefore, functional perturbation in Cx43 expression is an attractive candidate for dissecting the heterotypic interactions between HOBs and ECs. To further investigate this hypothesis, Cx43 was blocked (Cx43KO) by CRISPR in HOBs (Figure 6D). The Cx43KO HOBs were seeded in the 3D bicellular microfluidic device, and the endothelial leakiness was evaluated by dextran assay. Our results showed that loss of Cx43 in HOBs impacted the microvascular leakiness by ≈ 13.6 times (Figure 6E,F). Overall, we showed that HOBs impacted microvascular barrier function via Cx43‐mediated interactions with ECs.

We expanded our studies by evaluating the role of Cx43 in the heterotypic interactions under the presence of GCs. We investigated the impact of phosphorylated Cx43 expressed in HOBs on microvascular function. Phosphorylation mimic of the Cx43 S279/S282 (Cx43SD) increased the microvascular leakiness by ≈ 14 times, while mutations preventing phosphorylation of Cx43 S279/S282 (Cx43SA) in HOBs had no impact on the barrier function, indicating that dephosphorylation of S279/S282 in HOBs is necessary for bone microvascular barrier (Figure 6G,H). Finally, Cx43SA expressed in HOBs reversed the microvascular leakiness in the presence of GCs, indicating that dephosphorylation of Cx43 is essential and necessary for the heterotypic interactions and microvascular function (Figure 6I,J).

## 3 Discussion

Bone homeostasis is mediated by the function of microvasculature, osteoblasts, osteoclasts, osteocytes, and nerve cells within the extracellular matrix. Bone microvasculature is a complex microvessel network which supplies the bone with nutrients, oxygen, growth factors. They are surrounding by osteoblasts, osteoclasts, and osteocytes and all of which play crucial roles in bone microvascular remodeling and homeostasis.^[^ ^31^ ^]^ Osteocytes, embedded in the lacunae of the mineralized bone matrix, act as mechanosensory cells, regulating the biochemical signals of osteoblast and osteoclast activity.^[^ ^39^, ^40^ ^]^ They were traditionally thought to be terminally differentiated cells derived from mature osteoblasts. During this differentiation process, the expression of osteoblast markers, such as alkaline phosphatase, decreased, while the expression of osteocyte‐specific markers such as sclerostin and fibroblast growth factor 23 increased.^[^ ^41^ ^]^ Though osteoblasts and osteocytes originate from the same lineage, they serve distinct, vital functions in bone. Osteocytes are primarily responsible for maintaining bone homeostasis, while osteoblasts drive bone formation by synthesizing and mineralizing the bone matrix.^[^ ^42^ ^]^ Additionally, osteoblasts play a key role in bone microvascular regeneration and homeostasis through mechanisms such as the regulation of VEGF and angiopoietin‐1 (Ang‐1).^[^ ^43^, ^44^ ^]^ Specifically, osteoblasts secrete paracrine VEGF, which binds to VEGF receptors on endothelial cells, promoting endothelial migration and tube formation. Given the significant role of osteoblasts in endothelial function, in our study, we are further investigating the mechanisms underlying the osteoblast‐endothelial coupling, particularly in disease states like osteoporosis.

Osteoporosis is generally characterized by an imbalance between osteoblast and osteoclast activity, leading to abnormal bone remodeling.^[^ ^45^ ^]^ Specifically, GCs have been shown to upregulate osteoclast activity while downregulating osteoblast function, disrupting the delicate balance between these cell types, which is essential for maintaining bone mass.^[^ ^46^, ^47^ ^]^ GCs promote hyperactive osteoclasts^[^ ^48^ ^]^ and inflammation‐induced osteoclast activity,^[^ ^49^ ^]^ as well as an increase in empty lacunae.^[^ ^50^ ^]^ These findings are consistent with our hematoxylin and eosin (H&E) results, which showed a significant reduction in trabecular bone in Psl‐treated samples, suggesting increased osteoclast activity, while data from VK and AR assays indicated a loss of osteoblast activity. Osteoporosis is also associated with bone endothelial dysfunction. In postmenopausal osteoporosis, there is a reduction in sinusoidal and arterial capillaries within the bone marrow, leading to impaired blood perfusion. Additionally, studies have shown that elderly women with osteoporosis experience reduced bone blood flow in the femur.^[^ ^51^ ^]^ In line with these observations, our in vivo data demonstrated fewer and smaller blood vessels in Psl‐treated bone tissues, highlighting the impact of GCs on blood vessel formation in osteoporosis. These results underscore the multifaceted effects of GCs, particularly their influence on osteoclasts, osteoblasts, endothelial cells, and the overall bone microenvironment.^[^ ^52^ ^]^ To better understand this complexity, our study aims to investigate the mechanisms by which GCs regulate bone microvascular function, focusing on novel endothelial‐osteoblast interactions. Future research will explore the role of osteoclasts in these cellular interactions, providing a more comprehensive view of the GC‐induced disruptions in bone health.

One potential challenge to recapitulate this 3D bicellular interaction is that existing in vivo models are not suitable for studying rapid structural and functional changes of this coupling. Additionally, most existing in vitro models fail to recapitulate endothelial‐bone interactions in healthy and diseased states because they lack three‐dimensional (3D) vascular biomimetic architecture and shear stress.^[^ ^53^, ^54^, ^55^, ^56^, ^57^, ^58^ ^]^ To overcome these limitations, we engineered and utilized a 3D microfluidic bicellular system based on organ‐on‐a‐chip technology. Those platforms may assist in the identification of etiologic drivers of bone anomalies that can be targeted for rescue, which will benefit the development of novel personalized treatment decisions.^[^ ^59^, ^60^ ^]^

Specifically, our system consists of a fully perfusable microvessel surrounded by osteoblasts embedded in the extracellular matrix. We utilized the 3D endo‐osteo platform to measure microvascular integrity and osteoblast functionality in normal and GCs‐induced osteoporotic conditions. Specifically, by using this platform, we were able to measure the mineralization potential of osteoblasts by Osteoimage mineralization assay of the microvascular barrier for 18 days. Interestingly, we demonstrated that osteoblast enhanced microvascular barrier function, indicating the osteoblast contribution to bone homeostasis by regulating the mineralization process and microvascular function.

While a direct application of this model could involve diagnostics and the development of personalized treatments, our study aimed to investigate the pathogenic mechanisms underlying GC‐induced osteoporosis. Several studies have highlighted that Cx43 is a critical component of the intracellular machinery responsible for signal transduction in bone cells in response to pharmacologic, hormonal, and mechanical stimuli.^[^ ^61^, ^62^, ^63^ ^]^ Cx43 is highly expressed in bone cells, including osteoblasts, osteocytes, and osteoclasts, and is essential for their differentiation and function. Notably, alterations in Cx43 have been associated with the development of osteoporosis.^[^ ^64^, ^65^, ^66^ ^]^ Particular emphasis of our studies is focused on Cx43, which has been shown that plays a key role in endothelial intracellular communication and migration during angiogenesis.^[^ ^67^, ^68^, ^69^, ^70^ ^]^ Our in vivo results showed a loss of Cx43 membrane expression in the bone microvessels of Psl‐treated animals following 60 days of exposure. Additionally, our in vitro data revealed a decrease in Cx43 transcription levels after prolonged GC exposure (18 days). However, short‐term exposure to GCs (30 min, 60 min, and 120 min) did not affect total Cx43 protein levels,^[^ ^71^ ^]^ but it did increase Cx43 phosphorylation at Ser 282. This suggests a significant role of Cx43 in regulating bone microvascular function, particularly in the context of GC‐induced osteoporosis.

Although an extensive body of studies focuses on the Cx43 mechanism for each cell type, the adhesion and the mechanical cues mediated by the functional coupling between osteoblasts and endothelial cells and its effect on vascular function are less clear. Major challenges exist in identifying novel Cx43‐mediated heterotypic mechanisms based on the existing in vivo and in vitro models. Cx43 knockout mice are embryonic lethal, and it is difficult to identify the role of Cx43 in bone microvasculature.^[^ ^72^ ^]^ Additionally, using conditional knockout mice may be a laborious task,^[^ ^73^ ^]^ and current in vitro models struggle to identify new Cx43‐mediated bone microvascular barrier targets due to the lack of 3D vascular architecture and multi‐cellular compartments. Herein, using the 3D microfluidic platform, we demonstrated that Cx43 plays a key role in regulating endothelial cell‐osteoblast coupling and microvascular function. Our RT‐qPCR results showed that ZO‐1 and VE‐cadherin are primarily expressed in endothelial cells, with low transcription levels in osteoblasts, suggesting that these molecules are crucial for endothelial cell‐cell interactions rather than for interactions between endothelial cells and osteoblasts. In contrast, Cx43 is expressed in both endothelial cells and osteoblasts, highlighting its importance in regulating heterotypic endo‐osteoblast interactions, potentially through the MAPK pathway. Loss of Cx43 in osteoblasts resulted in the loss of endothelial osteoblast coupling and an increase in microvascular leakiness, indicating that Cx43 expressed in osteoblasts is essential for bone microvascular function. Notably, our results showed that GCs regulate the phosphorylation of Cx43 at Ser 282,^[^ ^74^ ^]^ suggesting activation of the MAPK pathway and subsequent internalization of Cx43. Moreover, we demonstrated that these phosphorylation sites are essential for mediating endo‐osteoblast interactions, as overexpressing these sites in osteoblasts led to a loss of barrier function. Interestingly, dephosphorylation of Cx43 in osteoblasts reversed this effect in the presence of GCs, reinforcing the idea that Cx43 is both essential and necessary for maintaining endothelial‐osteoblast coupling.

Overall, our study provides a deeper understanding of bone microvascular function by unraveling the complex dynamic interactions between endothelial cells and osteoblasts. To achieve this, we developed a robust on‐chip system capable of replicating the structures, mechanics, and intricate heterotypic interactions found in vivo. Future research will focus on integrating additional cell types, such as osteoclasts, to further explore their contribution on bone endothelium in normal and disease states.

## 4 Experimental Section

### Cell Culture and Reagents

Human osteoblasts (HOBs) (PromoCell) were cultured in osteoblast growth medium (PromoCell) (GM) and differentiated in osteoblast mineralization medium (MM) (PromoCell). Human umbilical vein endothelial cells (ECs) (Lonza) were cultured in endothelial cell growth medium EGM‐2 (Lonza). All experiments were performed with HOBs and ECs in passages 3 to 5. To achieve labeled HOBs labeled with a green fluorescent protein and ECs labeled red with mApple fluorescent protein, the cells were transduced with a lentiviral construct pCSCG‐EGFP and pCSCG‐mApple (Addgene), respectively, as described previously.^[^ ^32^ ^]^ Finally, to obtain Cx43 wild‐type (Cx43WT), Cx43 knockout (Cx43KO), Cx43‐S279/S282A (Cx43SA), and Cx43‐S279/S282D (Cx43SD) HOBs, the cells were transduced with LXSN‐Cx43, pLentiCRISPRv2 construct (gRNA sequencing: TCAGCGCACCACTGGTCGCA) (GeneScript), LXSN‐Cx43‐S279/S282A, and LXSN‐Cx43‐S279/S282D (Addgene), respectively.

### Fabrication of Microfluidic Platform

Scaffolds for the microfluidic devices were created as described previously.^[^ ^33^ ^]^ Polydimethylsiloxane (PDMS; Sylgard 184, Dow‐Corning; Krayden) devices were fabricated from these scaffolds. The PDMS devices were treated with 0.01% by volume fraction poly‐L‐lysine (PLL; Sigma) and 0.5% by volume fraction glutaraldehyde (Sigma). After washing overnight in water, steel acupuncture needles (diameter = 250 µm Seirin, Kyoto, Japan) were introduced into the devices. Next, 8 µg mL^−1^ fibrinogen (Sigma), 1 µg mL^−1^ thrombin (Sigma), and 1 mg mL^−1^ collagen I (BD) were mixed with 1 million HOBs, which were incubated for 18 days in MM. For the GC treatment, the HOBs were incubated for 18 days in MM in the presence of 200 nmol L^−1^ Hyd (Sigma), 100 nmol L^−1^ Pred (Sigma), and 100 nmol L^−1^ Psl (Sigma) in devices. Next, steel acupuncture needles(diameter = 250 µm) were removed, and ECs at a concentration of 10^6^ cells mL^−1^ were seeded into the channel. For the GC treatment, ECs were pre‐treated with Hyd (200 nmol L^−1^), Pred (100 nmol L^−1^), and Psl (100 nmol L^−1^) for 2 days prior to seeding into the devices. The devices were inverted to allow ECs to adhere to the top surfaces of the channel for 2 min and then flipped upright to allow ECs to adhere to the bottom surface of the channel for another 2 min. ECs that adhered to the fluid reservoirs were scraped off with a pipette tip, unattached cells in the channel were thoroughly flushed out with fresh EGM‐2 media, and the devices were placed on a platform rocker (BenchRocker BR2000) at 37 °C, CO_2_ incubator. Cells were cultured in the devices for 2 days prior to further analysis.

### Measurement of Endothelial Barrier

To measure the permeability of the endothelium in the microfluidic platform, fluorescent dextran (70 kDa Texas Red, Thermo Fisher) was introduced into perfusion media (EGM‐2) at a concentration of 12.5 µg mL^−1^. Diffusion of the dextran was imaged in real‐time with a confocal microscope (LSM 800, Carl Zeiss) at 10× magnification. An image time sequence was analyzed by taking the mean intensity over regions next to the cell layer in successive images. The time derivative of the intensity was determined by linear regression for each region. The time derivative of the intensity, the mean intensity (I), and the capillary radius (r) were used to determine the diffusive permeability coefficient (*P_d_*) by the equation $=\frac{dI}{dt}\;\times \frac{r}{2I}$. The uncertainty was determined by the standard deviation of the measured permeability at the different locations as described previously.^[^ ^32^ ^]^

### Osteogenesis Assay

The HOBs were seeded onto 48‐well culture plates at a density of 2×10^4^ cells/well and cultured in a GM or MM. For the GC treatment, the HOBs were incubated in MM in the presence of 200 nmol L^−1^ Hyd (Sigma), 100 nmol L^−1^ Pred (Sigma), and 100 nmol L^−1^ Psl (Sigma) for 1 week to 4 weeks. The medium was changed twice a week. The cells were fixed in 4 g paraformaldehyde (Sigma) per 100 ml phosphate‐buffered salin (PBS) for 20 min and washed twice in PBS. Osteoblast differentiation and mineralization were determined by detecting alkaline phosphatase (ALP; Leukocyte Alkaline Phosphatase Kit, Sigma‐Aldrich), Alizarin red (AR; Sigma‐Aldrich), and Von Kossa (VK; Sigma‐Aldrich) staining according to the protocols of the manufacturer and as described previously.^[^ ^75^, ^76^ ^]^ For the hydroxyapatite (HA) formation, HOBs in the devices were assessed using the Osteoimage mineralization assay kit (Lonza), as described previously.^[^ ^77^ ^]^ The mineralization potential of HOBs was imaged using a confocal microscope (LSM 800, Carl Zeiss), and image analysis for green, fluorescent HA formation was performed by ImageJ by performing a maximum intensity z projection and merging the channels.^[^ ^78^ ^]^

### Immunofluorescence Staining

For immunofluorescence staining, cells in the devices were fixed in 4 g paraformaldehyde (Sigma; St, Louis, MI, USA) per 100 mL in PBS for 30 min and washed twice in PBS. For Cx43 labeling, cells were permeabilized with 0.1% (by volume fraction) Triton‐X 100 in PBS for 30 min and treated with blocking solution (0.01% (by volume fraction) Triton‐X 100, 5 g/100 mL goat serum (Sigma) in PBS) overnight at 4 °C and washed three times with PBS. Primary antibody against rabbit anti‐human Cx43 (1:100 dilution in blocking solution; Cell Signaling Technology) was incubated overnight at 4 °C. Secondary antibodies (1:100 dilution in blocking solution, Alexa 568‐conjugated goat‐anti‐rabbit IgG; Invitrogen) were incubated overnight at 4 °C. Cell nuclei were labeled with DAPI (1:300 dilution in blocking solution; Sigma). Cell in devices were imaged using confocal microscope (LSM 800, Carl Zeiss), and ImageJ was used to perform z‐projection for all stacks, merge channels, and generate longitudinal and transverse cross‐sections.^[^ ^78^ ^]^

### Enzyme‐Linked Immunosorbent Assay (ELISA)

Protein levels of the secreted Pro‐Collagen I alpha 1 (Pro‐COLA1) in cell culture supernatants were measured using a human Pro‐Collagen I α1‐specific sandwich ELISA system (R&D Systems) according to the manufacturer's manual. Serially diluted recombinant human Pro‐Collagen I α1 was used as the standard. All samples in each analysis were examined in triplicate.

### Western Blot Analysis

For Western Blot analysis, HOBs and ECs were co‐plated at a density of 3000 cells/cm^2^ and 30000 cells/cm^2^, respectively. The cells were cultured in the presence of 200 nmol L^−1^ Hyd (Sigma), 100 nmol L^−1^ Pred (Sigma), and 100 nmol L^−1^ Psl (Sigma) for 30 min, 60 min, and 120 min. Also, the co‐culture was incubated in the presence of 200 nmol L^−1^ Hyd (Sigma), 100 nmol L^−1^ Pred (Sigma), and 100 nmol L^−1^ Psl (Sigma) and an inhibitors cocktail for MAPK/ERK pathway, consisting of 25 µmol L^−1^ SP600125, 10 µmol L^−1^ SCH772984, 30   µmol L^−1^ Losmapimod, as reported previously,^[^ ^79^, ^80^, ^81^ ^]^ for 30 min. The cells were washed with ice‐cold PBS and lysed in RIPA buffer (Thermo Scientific) containing protease and phosphatase inhibitor cocktails (Cell Signaling). Cell lysates were centrifuged at 12000 g for 10 min at 4 °C, the supernatants were collected, and the proteins were resolved in 4% to 12% gradient SDS–PAGE gels. Proteins were transferred to nitrocellulose membranes (Thermo Fisher) and were probed with the following antibodies: rabbit‐anti human phospho‐p44/42 MAPK (ERK1/2) (1:1000; Cell Signaling; Cat number: 9101); rabbit‐anti human p44/42 MAPK (ERK1/2) (1:1000; Cell Signaling; Cat number: 9102); rabbit‐anti human phospho‐p38 MAPK (1:1000; Cell Signaling; Cat number: 9211); rabbit‐anti human p38 MAPK (Thr180/Tyr182) (1:1000; Cell Signaling; Cat number: 9212); rabbit anti‐human phospho‐ SAPK/JNK (Thr183/185) (1:1000; Cell Signaling; Cat number: 9251); rabbit anti‐human SAPK/JNK (1:1000; Cell Signaling; Cat number: 9252); phospho Ser282 Cx43 (1:500; Thermofisher; Cat number: PA5‐105957); Cx43 (1:1000; Cell Signaling; Cat number: 3512S); β‐actin (1:1000; Cell Signaling; Cat number: 4967). The antibodies were diluted and incubated in 5 g BSA per 100 mL Tris‐buffered saline with 0.1% (by volume fraction) Tween 20 Detergent (TBST); Thermo Fisher) overnight in 4 °C. Proteins of interest were detected with horseradish peroxidase‐conjugated secondary antibodies (anti‐rabbit IgG, HRP‐linked Antibody 1:1000 dilution; Cell Signaling; Cat number: 7074) and visualized with the chemiluminescent substrate (Thermo Scientific), according to manufacturer instructions.^[^ ^75^ ^]^ All western blots were quantified by Image J according to standard procedures.^[^ ^78^ ^]^

### Real‐Time Quantitative PCR Analysis

Total RNA was isolated from the 3D platform using TRIzol Reagent (Invitrogen, Carlsbad, CA) following the manufacturer's instruction. SuperScript III cDNA Synthesis kit (Thermo Fisher Scientific) was used to synthesize cDNA from RNA in Applied Biosystems Veriti 96‐Well Thermal Cycler. The real‐time quantitative PCR (RT‐qPCR) was performed using a PowerTrack SYBR Green Master Mix and ViiA 7 Real‐Time PCR System with 96‐Well Block (Applied Biosystems, USA) in accordance with the manufacturer's instructions. The primers used in this study are described in **Table** **1**. The expression level of each gene was normalized to the expression level of 18S ribosomal RNA (18S rRNA) internal control. The relative gene expression was calculated by the standard curve method using the target Cq values and the Cq value for 18S rRNA.

**Table 1:** PCR primers used in this study.

Gene name	Primer sequences	
human ZO‐1	Forward	5′‐GTC CAG AAT CTC GGA AAA GTG CC‐3′
	Reverse	5′‐CTT TCA GCG CAC CAT ACC AAC C‐3′
human CDH5	Forward	5′‐AAG CGT GAG TCG CAA GAA TG‐3′
	Reverse	5′‐TCT CCA GGT TTT CGC CAG TG‐3′
human Cx43	Forward	5′‐TGGTAAGGTGAAAATGCGAGG‐3′
	Reverse	5′‐GCACTCAAGCTGAATCCATAGAT‐3′
human Runx2	Forward	5′‐TGG TTA CTG TCA TGG CGG GTA‐3′
	Reverse	5′‐TCT CAG ATC GTT GAA CCT TGC TA‐3′
human ColA1	Forward	5′‐GGC CCT CAA GGT TTC CAA GG‐3′
	Reverse	5′‐CAC CCT GTG GTC CAA CAA CTC‐3′
human IntB1	Forward	5′‐GGA TTC TCC AGA AGG TGG TTT CG‐3′
	Reverse	5′‐TGC CAC CAA GTT TCC CAT CTC C‐3′
human 18S	Forward	5′‐CGG CTA CCA CAT CCA AGG AA‐3′
	Reverse	5′‐GCT GGA ATT ACC GCG GCT‐3′

Abbreviation: ZO‐1, Zonula occludens‐1 (Tight junction protein‐1); CDH5, VE‐cad (vascular endothelial cadherin); Cx43, Connexin43; Runx2, Runt‐related transcription factor 2; ColA1, Collagen type 1 alpha 1; IntB1, Integrin beta‐1; 18S, 18S ribosomal RNA.

### In Vivo Studies

All procedures involved in animal experiments (IACUC Protocol Number: 2022‐0058‐ Title: Effect of glucocorticoids on bone vascularization) were approved by the Georgetown University Institutional Animal Care and Use Committee. 4 months to 6 months‐old Swiss Webster mice were purchased from Charles River Laboratories. The mice were randomized into two groups, 10 animals each, with an equal number of males and females. The GC‐treated group was implanted subcutaneously in the dorsal scapular region with slow‐release pellets containing Psl at the dose of 3.8 mg/60 days = 2.1 mg/kg/day, assuming 30 g as the average weight of this mouse strain (Innovative Research of America, Sarasota, FL). The control group received placebo pellets containing the corresponding vehicle. Throughout the experiment, mice were monitored for body weight loss. 60 days post pellet implantation, the mice were euthanized, the long bones harvested, fixed in 10% (per volume) neutral buffered formalin, decalcified in 14% (by volume fraction) EDTA, paraffin‐embedded, and subjected to histopathological analyses.

### Tissue Analysis

To examine the effect of GCs in mineralization, AR and VK stainings were performed according to the manufacturer's protocols and as described previously.^[^ ^75^, ^76^ ^]^ For immunofluorescence, the tissue sections were deparaffinized^[^ ^82^ ^]^ and were permeabilized with 0.1% (by volume fraction) Triton‐X 100 in PBS for 30 min and treated with blocking solution (0.01% (by volume fraction) Triton‐X 100, 5 g/100 mL goat serum (Sigma) in PBS) overnight at 4 °C and washed three times with PBS. Next, the tissues were incubated in Isolectin GS‐IB_4_ from *Griffonia simplicifolia*, Alexa Fluor 568 Conjugate (1:100; Thermofisher) and DAPI (1:300; Thermofisher). For Cx43 staining, the primary antibody against rabbit anti‐human Cx43 (1:100 dilution; Cell Signaling Technology) was incubated overnight at 4 °C. Next day, the tissue slides were washed twice in PBS, and a secondary antibody (1:100 dilution, Alexa 488‐conjugated goat‐anti‐rabbit IgG; Invitrogen) was incubated for 1 h at RT. Cell nuclei were labeled with DAPI (1:300 dilution; Thermofisher). Imaging was performed by confocal microscope Nikon CSU‐W1 SoRa Spinning Disk Microscope, and image analysis was performed by Image J.

### Statistical Analysis

Statistical analysis of the data was performed using one‐way analysis of variance (ANOVA), using Tukey post‐test for more than two variables. The *P*‐value was set to be significant if < 0.05 unless differently stated in the text. All results were expressed as mean plus or minus one standard deviation. In each test, the number of independent experiments (N) was more than three, and the number of data points (n) in each experiment was different. Both N and n were shown in the figure legends.

## Conflict of Interest

The authors declare no conflict of interest.

## Author Contributions

E.L. and S.A. conceptualized the idea for the study. E.L., P.L., M.C., P.S., Y.K., J.K., and S.A. designed the methodology. E.L., P.S., and S.A. developed the software. E.L. and S.A. performed the validation. S.A. acquired resources. E.L. and S.A. made the preparation for writing the original draft. E.L., P.L., M.C., J.W., Y.K., S.H., M.E., M.L., J.K., and S.A. wrote, reviewed, and edited the manuscript. All authors have read and agreed to the published version of the manuscript.

## Supporting information



Supporting Information

Supporting Information

Supporting Information

## Data Availability

The data that support the findings of this study are available from the corresponding author upon reasonable request.

## References

[1] Z. Chen , A. Bozec , A. Ramming , G. Schett , Nat. Rev. Rheumatol. 2019, 15, 9. 30341437 10.1038/s41584-018-0109-2

[2] L. Duan , X. Rao , K. R. Sigdel , J. Immunol. Res. 2019, 2019, 7403796. 30944837 10.1155/2019/7403796 PMC6421792

[3] E. Nikoopour , J. A. Schwartz , B. Singh , Inflamm. Allergy Drug Targets 2008, 7, 203. 18782028 10.2174/187152808785748155

[4] A. E. Coutinho , K. E. Chapman , Mol. Cell. Endocrinol. 2011, 335, 2. 20398732 10.1016/j.mce.2010.04.005 PMC3047790

[5] C. D. B. Maria , G. Petrillo , J. A. Cidlowski , in Book: The Hypothalamic‐Pituitary‐Adrenal Axis in Health and Disease (Ed: E. Geer ), Springer, Cham 2017, pp. 43.

[6] T. Rhen , J. A. Cidlowski , New Engl. J. Med. 2005, 353, 1711. 16236742 10.1056/NEJMra050541

[7] K. Briot , C. Roux , RMD Open 2015, 1, e000014. 26509049 10.1136/rmdopen-2014-000014 PMC4613168

[8] L. Buckley , M. B. Humphrey , New Engl. J. Med. 2018, 379, 2547. 30586507 10.1056/NEJMcp1800214

[9] J. Compston , Nat. Rev. Rheumatol. 2010, 6, 82. 20125175 10.1038/nrrheum.2009.259

[10] M. Luengo , C. Picado , L. Del Rio , N. Guanabens , J. M. Montserrat , J. Setoain , Thorax 1991, 46, 803. 1771602 10.1136/thx.46.11.803 PMC1021033

[11] E. Canalis , G. Mazziotti , A. Giustina , J. P. Bilezikian , Osteoporosis Int. 2007, 18, 1319. 10.1007/s00198-007-0394-0 17566815

[12] E. Canalis , Curr. Opin. Rheumatol. 2003, 15, 454. 12819474 10.1097/00002281-200307000-00013

[13] N. E. Lane , W. Yao , M. Balooch , R. K. Nalla , G. Balooch , S. Habelitz , J. H. Kinney , L. F. Bonewald , J. Bone Miner. Res. 2006, 21, 466. 16491295 10.1359/JBMR.051103 PMC1797152

[14] R. S. Weinstein , R. L. Jilka , A. M. Parfitt , S. C. Manolagas , J. Clin. Invest. 1998, 102, 274. 9664068 10.1172/JCI2799 PMC508885

[15] C. Swanson , M. Lorentzon , H. H. Conaway , U. H. Lerner , Endocrinology 2006, 147, 3613. 16614077 10.1210/en.2005-0717

[16] B. C. Silva , J. P. Bilezikian , Curr. Opin. Pharmacol. 2015, 22, 41. 25854704 10.1016/j.coph.2015.03.005 PMC5407089

[17] R. L. Jilka , C. A. O'Brien , S. M. Bartell , R. S. Weinstein , S. C. Manolagas , J. Bone Miner. Res. 2010, 25, 2427. 20533302 10.1002/jbmr.145 PMC3179285

[18] G. G. Walmsley , R. C. Ransom , E. R. Zielins , T. Leavitt , J. S. Flacco , M. S. Hu , A. S. Lee , M. T. Longaker , D. C. Wan , Stem Cell Rev. Rep. 2016, 12, 524. 27250635 10.1007/s12015-016-9665-5 PMC5053855

[19] Z. S. Ai‐Aql , A. S. Alagl , D. T. Graves , L. C. Gerstenfeld , T. A. Einhorn , J. Dental Res. 2008, 87, 107. 10.1177/154405910808700215 PMC3109437 18218835

[20] T. A. Einhorn , L. C. Gerstenfeld , Nat. Rev. Rheumatol. 2015, 11, 45. 25266456 10.1038/nrrheum.2014.164 PMC4464690

[21] J. Street , M. Bao , L. deGuzman , S. Bunting , F. V. Peale Jr. , N. Ferrara , H. Steinmetz , J. Hoeffel , J. L. Cleland , A. Daugherty , N. van Bruggen , H. P. Redmond , R. A. Carano , E. H. Filvaroff , Proc. Natl. Acad. Sci. USA 2002, 99, 9656. 12118119 10.1073/pnas.152324099 PMC124965

[22] T. Asahara , T. Takahashi , H. Masuda , C. Kalka , D. Chen , H. Iwaguro , Y. Inai , M. Silver , J. M. Isner , EMBO J. 1999, 18, 3964. 10406801 10.1093/emboj/18.14.3964 PMC1171472

[23] P. Guo , B. Hu , W. Gu , L. Xu , D. Wang , H. J. Huang , W. K. Cavenee , S. Y. Cheng , Am. J. Pathol. 2003, 162, 1083. 12651601 10.1016/S0002-9440(10)63905-3 PMC1851242

[24] J. Fiedler , G. Roderer , K. P. Gunther , R. E. Brenner , J. Cell. Biochem. 2002, 87, 305. 12397612 10.1002/jcb.10309

[25] M. Cashin , C. Coombs , I. Torode , J. Pediatr. Orthop. 2018, 38, e83. 29176457 10.1097/BPO.0000000000000866

[26] T. Fang , E. W. Zhang , F. C. Sailes , R. A. McGuire , W. C. Lineaweaver , F. Zhang , Arch. Orthop. Trauma Surg. 2013, 133, 1. 23076656 10.1007/s00402-012-1627-z

[27] D. S. Patel , M. Roth , N. Kapil , Am. Fam. Phys. 2011, 83, 39. 21888126

[28] B. Shadur , I. Zaidman , A. NaserEddin , E. Lokshin , F. Hussein , H. C. Oron , B. Avni , S. Grisariu , P. Stepensky , Pediatr. Blood Cancer 2018, 65, e27010. 29469225 10.1002/pbc.27010

[29] J. Filipowska , K. A. Tomaszewski , L. Niedzwiedzki , J. A. Walocha , T. Niedzwiedzki , Angiogenesis 2017, 20, 291. 28194536 10.1007/s10456-017-9541-1 PMC5511612

[30] E. C. Watson , R. H. Adams , Cold Spring Harbor Perspect. Med. 2018, 8. 10.1101/cshperspect.a031559 PMC6027931 28893838

[31] E. J. Lee , M. Jain , S. Alimperti , Tissue Eng. Part B, Rev. 2021, 27, 313. 32940150 10.1089/ten.teb.2020.0154 PMC8390780

[32] S. Alimperti , T. Mirabella , V. Bajaj , W. Polacheck , D. M. Pirone , J. Duffield , J. Eyckmans , R. K. Assoian , C. S. Chen , Proc. Natl. Acad. Sci. USA 2017, 114, 8758. 28765370 10.1073/pnas.1618333114 PMC5565405

[33] P. F. Salipante , S. D. Hudson , S. Alimperti , Soft Matter 2021, 18, 117. 34816867 10.1039/d1sm01312b PMC9001019

[34] D. Gonzalez‐Nieto , L. Li , A. Kohler , G. Ghiaur , E. Ishikawa , A. Sengupta , M. Madhu , J. L. Arnett , R. A. Santho , S. K. Dunn , G. I. Fishman , D. E. Gutstein , R. Civitelli , L. C. Barrio , M. Gunzer , J. A. Cancelas , Blood 2012, 119, 5144. 22498741 10.1182/blood-2011-07-368506 PMC3369607

[35] K. E. Johnson , S. Mitra , P. Katoch , L. S. Kelsey , K. R. Johnson , P. P. Mehta , Mol. Biol. Cell 2013, 24, 715. 23363606 10.1091/mbc.E12-07-0537 PMC3596244

[36] L. P. Latchford , L. S. Perez , J. E. Conage‐Pough , R. Turk , M. A. Cusimano , V. I. Vargas , S. Arora , S. R. Shienvold , R. R. Kulp , H. M. Belverio , F. M. White , A. F. Thevenin , The Journal of biological chemistry 2025, 108178. 39798878 10.1016/j.jbc.2025.108178 PMC11870265

[37] J. L. Solan , P. D. Lampe , Biochim. Biophys. Acta Biomembr. 1860, 2018, 83. 10.1016/j.bbamem.2017.04.008 PMC5640473 28414037

[38] L. Yang , G. Zhou , M. Li , Y. Li , L. Yang , Q. Fu , Y. Tian , Diabetes Metab. Syndr. Obes. 2020, 13, 545. 32161481 10.2147/DMSO.S239892 PMC7049751

[39] U. H. Lerner , Semin. Orthod. 2012, 18, 237.

[40] L. Qin , W. Liu , H. Cao , G. Xiao , Bone Res. 2020, 8, 23. 32550039 10.1038/s41413-020-0099-y PMC7280204

[41] Y. Kitase , M. Prideaux , Bone 2023, 170, 116724. 36868508 10.1016/j.bone.2023.116724 PMC10062476

[42] T. Mizoguchi , N. Ono , J. Bone Miner. Res. 2021, 36, 1432. 34213032 10.1002/jbmr.4410 PMC8338797

[43] F. Diomede , G. D. Marconi , L. Fonticoli , J. Pizzicanella , I. Merciaro , P. Bramanti , E. Mazzon , O. Trubiani , Int. J. Mol. Sci. 2020, 21, 3242. 32375269 10.3390/ijms21093242 PMC7247346

[44] K. Hu , B. R. Olsen , Bone 2016, 91, 30. 27353702 10.1016/j.bone.2016.06.013 PMC4996701

[45] X. Feng , J. M. McDonald , Annu. Rev. Pathol. 2011, 6, 121. 20936937 10.1146/annurev-pathol-011110-130203 PMC3571087

[46] C. A. O'Brien , D. Jia , L. I. Plotkin , T. Bellido , C. C. Powers , S. A. Stewart , S. C. Manolagas , R. S. Weinstein , Endocrinology 2004, 145, 1835. 14691012 10.1210/en.2003-0990

[47] D. Jia , C. A. O'Brien , S. A. Stewart , S. C. Manolagas , R. S. Weinstein , Endocrinology 2006, 147, 5592. 16935844 10.1210/en.2006-0459 PMC1819400

[48] K. Chen , Y. Liu , J. He , N. Pavlos , C. Wang , J. Kenny , J. Yuan , Q. Zhang , J. Xu , W. He , Int. J. Biol. Sci. 2020, 16, 1888. 32398957 10.7150/ijbs.40917 PMC7211180

[49] D. Ngo , E. Beaulieu , R. Gu , A. Leaney , L. Santos , H. Fan , Y. Yang , W. Kao , J. Xu , V. Escriou , S. Loiler , M. J. Vervoordeldonk , E. F. Morand , Arthritis Rheum. 2013, 65, 1203. 23335223 10.1002/art.37858

[50] G. Wang , C. Ma , L. Mo , J. Chen , J. Yuan , J. Xu , W. He , J. Orthop. Trans. 2024, 45, 178. 10.1016/j.jot.2024.01.009 PMC10973539 38549807

[51] K. Alagiakrishnan , A. Juby , D. Hanley , W. Tymchak , A. Sclater , J. Gerontol. Ser. A, Biol. Sci. Med. Sci. 2003, 58, 362. 12663699 10.1093/gerona/58.4.m362

[52] X. Liu , Y. Chai , G. Liu , W. Su , Q. Guo , X. Lv , P. Gao , B. Yu , G. Ferbeyre , X. Cao , M. Wan , Nat. Commun. 2021, 12, 1832. 33758201 10.1038/s41467-021-22131-1 PMC7987975

[53] J. R. Shapiro , D. J. McBride Jr. , N. S. Fedarko , Connect. Tissue Res. 1995, 31, 265. 15612365 10.3109/03008209509010820

[54] A. S. Kamoun‐Goldrat , M. F. Le Merrer , J. Bone Miner. Metab. 2007, 25, 211. 17593490 10.1007/s00774-007-0750-3

[55] I. Grafe , T. Yang , S. Alexander , E. P. Homan , C. Lietman , M. M. Jiang , T. Bertin , E. Munivez , Y. Chen , B. Dawson , Y. Ishikawa , M. A. Weis , T. K. Sampath , C. Ambrose , D. Eyre , H. P. Bachinger , B. Lee , Nat. Med. 2014, 20, 670. 24793237 10.1038/nm.3544 PMC4048326

[56] S. D. Chipman , H. O. Sweet , D. J. McBride Jr. , M. T. Davisson , S. C. Marks Jr. , A. R. Shuldiner , R. J. Wenstrup , D. W. Rowe , J. R. Shapiro , Proc. Natl. Acad. Sci. USA 1993, 90, 1701. 8446583 10.1073/pnas.90.5.1701 PMC45947

[57] J. Ma , J. J. van den Beucken , F. Yang , S. K. Both , F. Z. Cui , J. Pan , J. A. Jansen , Tissue Eng. Part C, Methods 2011, 17, 349. 20932081 10.1089/ten.TEC.2010.0215

[58] S. Shafiee , S. Shariatzadeh , A. Zafari , A. Majd , H. Niknejad , Front. Bioeng. Biotechnol. 2021, 9, 745314. 34900955 10.3389/fbioe.2021.745314 PMC8655789

[59] D. E. Ingber , Nat. Rev. Genet. 2022, 23, 467. 35338360 10.1038/s41576-022-00466-9 PMC8951665

[60] S. N. Bhatia , D. E. Ingber , Nat. Biotechnol. 2014, 32, 760. 25093883 10.1038/nbt.2989

[61] L. I. Plotkin , T. Bellido , BoneBone 2013, 52, 157. 10.1016/j.bone.2012.09.030 PMC3513515 23041511

[62] L. I. Plotkin , T. L. Speacht , H. J. Donahue , Curr. Osteoporos. Rep. 2015, 13, 67. 25616771 10.1007/s11914-015-0255-2 PMC4355098

[63] T. Zappitelli , J. E. Aubin , J. Cell. Biochem. 2014, 115, 1646. 24818806 10.1002/jcb.24836

[64] L. Ma , R. Hua , Y. Tian , H. Cheng , R. J. Fajardo , J. J. Pearson , T. Guda , D. B. Shropshire , S. Gu , J. X. Jiang , Bone Res. 2019, 7, 11. 31016065 10.1038/s41413-019-0050-2 PMC6476886

[65] D. Zhang , X. Li , C. Pi , L. Cai , Y. Liu , W. Du , W. Yang , J. Xie , Acta Biochim. Biophys. Sin. 2020, 52, 517. 32286624 10.1093/abbs/gmaa025

[66] D. Zhao , R. Liu , G. Li , M. Chen , P. Shang , H. Yang , J. X. Jiang , H. Xu , Front. Physiol. 2020, 11, 299. 32296345 10.3389/fphys.2020.00299 PMC7137730

[67] J. J. O'Donnell 3rd , A. A. Birukova , E. C. Beyer , K. G. Birukov , PLoS One 2014, 9, e100931. 24967639 10.1371/journal.pone.0100931 PMC4072707

[68] R. E. Strauss , L. Mezache , R. Veeraraghavan , R. G. Gourdie , Biomolecules 2021, 11, 1192. 34439858 10.3390/biom11081192 PMC8393261

[69] H. H. Wang , C. H. Su , Y. J. Wu , J. Y. Li , Y. M. Tseng , Y. C. Lin , C. L. Hsieh , C. H. Tsai , H. I. Yeh , Angiogenesis 2013, 16, 553. 23354732 10.1007/s10456-013-9335-z

[70] H. Mannell , P. Kameritsch , H. Beck , A. Pfeifer , U. Pohl , K. Pogoda , Int. J. Mol. Sci. 2021, 23, 294. 35008716 10.3390/ijms23010294 PMC8745637

[71] C. Shen , M. R. Kim , J. M. Noh , S. J. Kim , S. O. Ka , J. H. Kim , B. H. Park , J. H. Park , Calcif. Tissue Int. 2016, 99, 88. 26914606 10.1007/s00223-016-0121-y

[72] R. Dermietzel , Y. Gao , E. Scemes , D. Vieira , M. Urban , M. Kremer , M. V. Bennett , D. C. Spray , Brain Res. 2000, 32, 45. 10.1016/s0165-0173(99)00067-3 10751656

[73] M. Kadowaki , S. Nakamura , O. Machon , S. Krauss , G. L. Radice , M. Takeichi , Dev. Biol. 2007, 304, 22. 17222817 10.1016/j.ydbio.2006.12.014

[74] J. L. Solan , P. D. Lampe , Biochem. J. 2009, 419, 261. 19309313 10.1042/BJ20082319 PMC2669545

[75] E. J. Lee , S. M. Kim , B. Choi , E. Y. Kim , Y. H. Chung , E. J. Lee , B. Yoo , C. K. Lee , S. Hong , B. J. Kim , J. M. Koh , S. H. Kim , Y. G. Kim , E. J. Chang , Sci. Rep. 2017, 7, 40240. 28079119 10.1038/srep40240 PMC5228062

[76] E. J. Lee , E. J. Lee , Y. H. Chung , D. H. Song , S. Hong , C. K. Lee , B. Yoo , T. H. Kim , Y. S. Park , S. H. Kim , E. J. Chang , Y. G. Kim , Arthritis Res. Ther. 2015, 17, 350. 26634249 10.1186/s13075-015-0870-4 PMC4669668

[77] Y. Kim , E. J. Lee , A. V. Davydov , S. Frukhtbeyen , J. E. Seppala , S. Takagi , L. Chow , S. Alimperti , Biomed. Mater. 2021, 16, 045002. 10.1088/1748-605X/abcf03 PMC8164647 33254152

[78] C. A. Schneider , W. S. Rasband , K. W. Eliceiri , Nat. Methods 2012, 9, 671. 22930834 10.1038/nmeth.2089 PMC5554542

[79] K. Burkhard , P. Shapiro , Methods Mol. Biol. 2010, 661, 107. 20811979 10.1007/978-1-60761-795-2_6 PMC3727897

[80] A. Chaikuad , E. M. Tacconi , J. Zimmer , Y. Liang , N. S. Gray , M. Tarsounas , S. Knapp , Nat. Chem. Biol. 2014, 10, 853. 25195011 10.1038/nchembio.1629 PMC4687050

[81] L. K. Newby , M. S. Marber , C. Melloni , L. Sarov‐Blat , L. H. Aberle , P. E. Aylward , G. Cai , R. J. de Winter , C. W. Hamm , J. F. Heitner , R. Kim , A. Lerman , M. R. Patel , J. F. Tanguay , J. J. Lepore , H. R. Al‐Khalidi , D. L. Sprecher , C. B. Granger , S. Investigators , Lancet 2014, 384, 1187. 24930728 10.1016/S0140-6736(14)60417-7

[82] P. F. Marinho , T. Hanscheid , MethodsX 2023, 10, 102079. 36865652 10.1016/j.mex.2023.102079 PMC9971263

