# Supplementary material for: Glucocorticoids Alter Bone Microvascular Barrier via MAPK/Connexin43 Mechanisms
Source: Adv Healthc Mater. 2025 Jan 20;14(7):2404302. doi: 10.1002/adhm.202404302 (PMC11912118; doi:10.1002/adhm.202404302)
Supplement: Supplementary file 1 — Supporting Information [file ADHM-14-0-s002.docx]

Supporting Information

Title: Glucocorticoids Alter Bone Microvascular Barrier via MAPK/Connexin43 Mechanisms

Running title: Connexin43 Mechanisms Mediate Osteoblast-Endothelial Interaction

Eun-Jin Lee, Peter Lialios, Micaila Curtis, James Williams IV, Yoontae Kim, Paul Salipante, Steven Hudson, Mandy Esch, Moshe Levi, Joanna Kitlinska, Stella Alimperti*

**
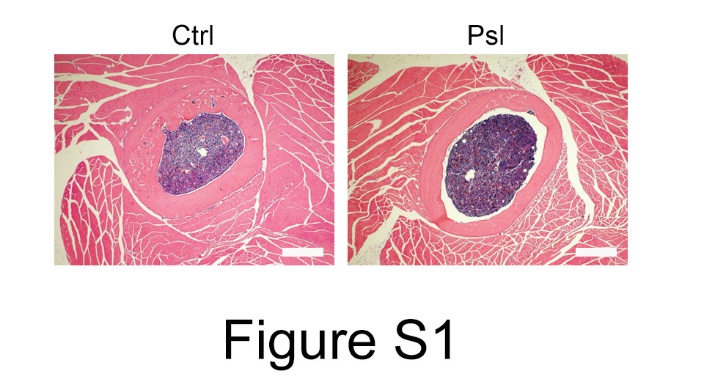
**

**Figure Supplementary 1.** Hematoxylin and eosin (H&E) staining of hind limb sections from mouse implanted with prednisolone (Psl) pellets for 60 days. Mouse hind limbs were dissected, fixed, and decalcified, and the cross sections with cortical region. Scale bars, 500 µm.


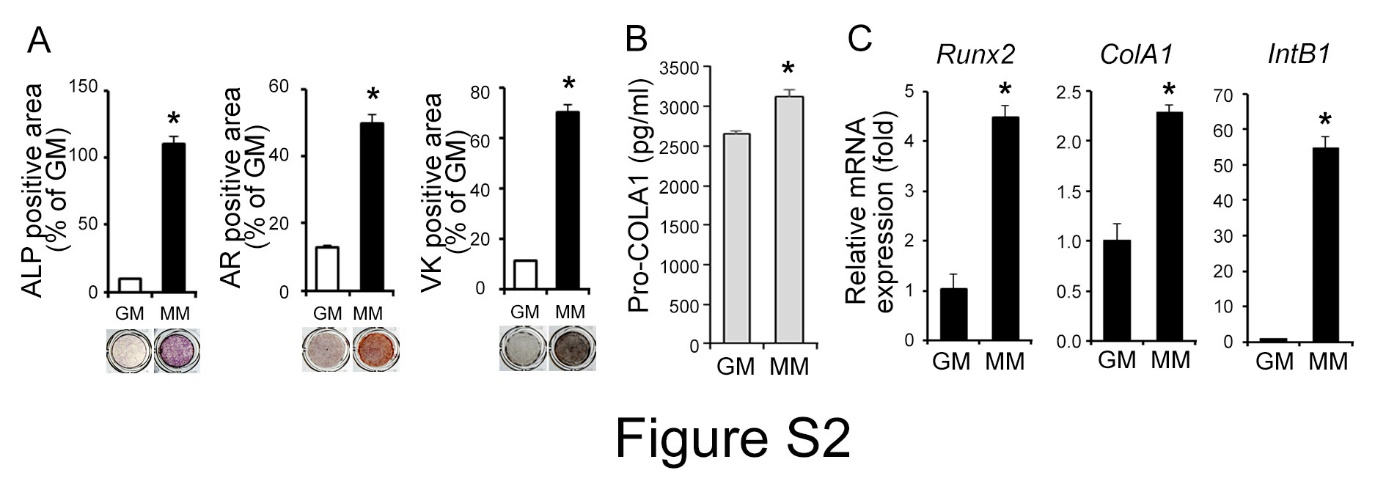


**Figure Supplementary 2.** Optimization of mineralization conditions. A) HOBs were cultured in 48 well plates for 3 weeks with osteoblast growth media (GM) and osteoblast mineralization media (MM). The cells were used for alkaline phosphatase (ALP), Alizarin red (AR), and Von Kossa (VK) staining. The bar graph presents the ALP-positive area, AR-positive area, and VK-positive area measured in each cultured dish (upper panel). N = 3. B) Culture supernatants were collected, and collagen type 1 protein levels were analyzed by ELISA. N = 3. C) RNA was isolated from 3D devices (18 d mineralization). The expression of osteoblast-specific genes was examined by RT-qPCR. The transcript levels were normalized to 18S. The quantitative data are expressed as means ± SD. N= 3; significant differences: **, p-value* < 0.05.


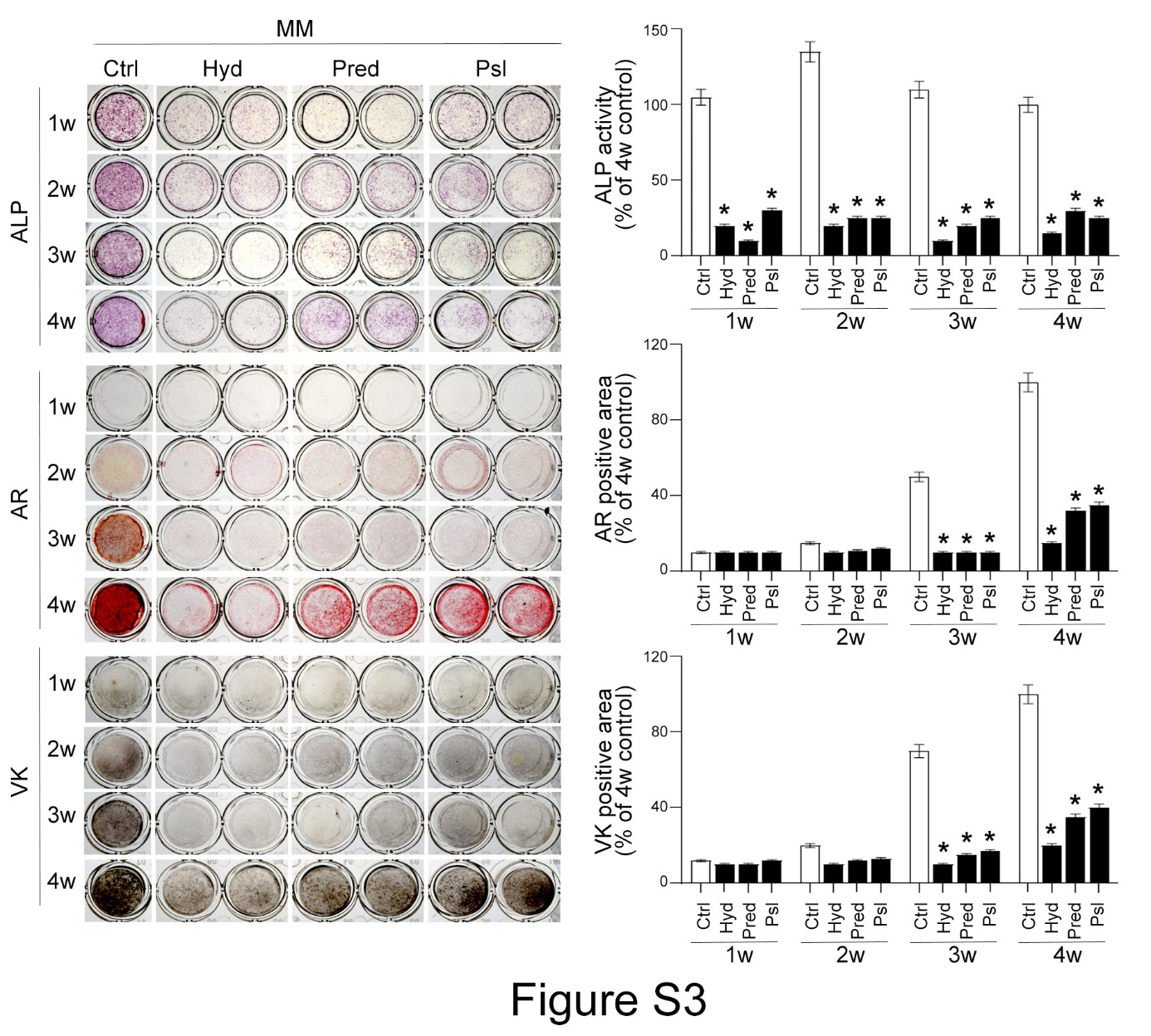


**Figure Supplementary 3.** GCs downregulate osteogenic potential. HOBs were cultured in 48 well plates for 1 week to 4 weeks with osteogenic medium in the presence of GCs such as Hyd, Pred, and Psl. The cells were used for alkaline phosphatase (ALP), Alizarin red (AR), and Von Kossa (VK) staining. The bar graph presents the ALP-positive area, AR-positive area, and VK-positive area measured in each cultured dish (right panel). The quantitative data are expressed as means ± SD. N= 3; significant differences: **, p-value* < 0.05.


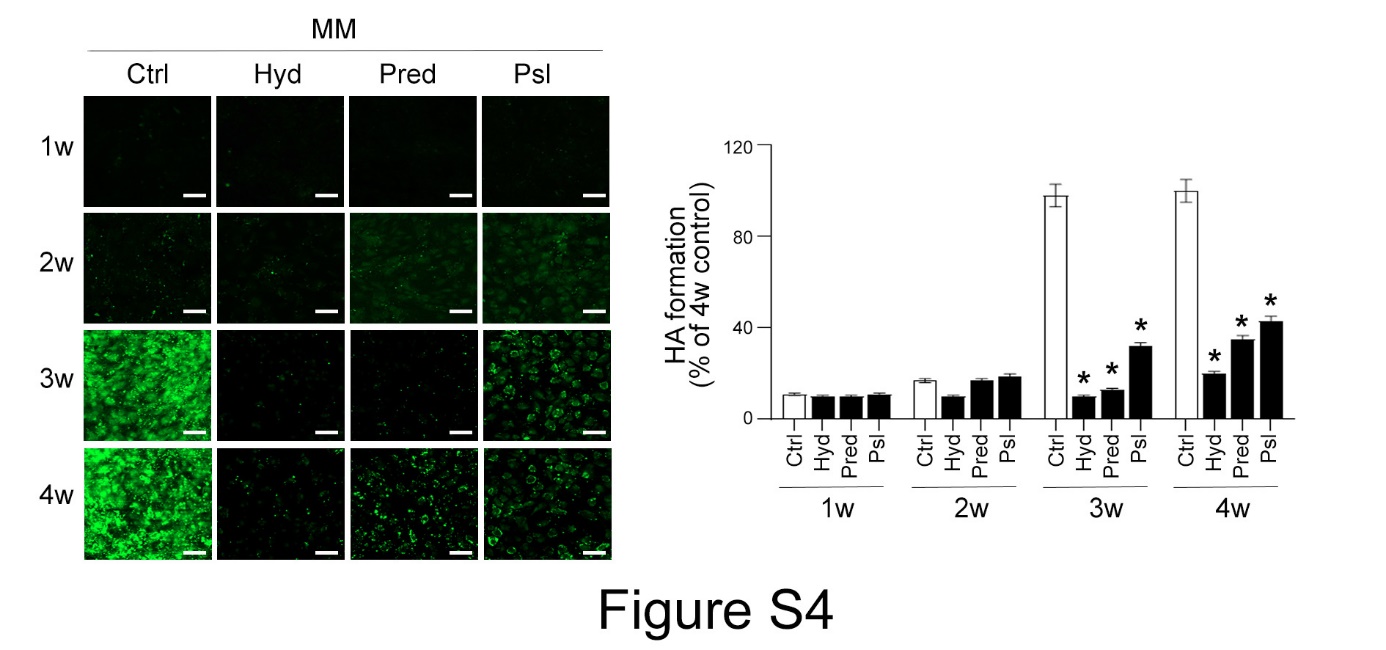


**Figure Supplementary 4.** GCs inhibit HA formation. HOBs were cultured in 48 well plates for 1 week to 4 weeks with the osteogenic medium in the presence of GCs such as Hyd, Pred, and Psl. The cells were stained for osteoimage assay. Scale bars, 200 µm. The bar graph presents the measured HA (green) in each cultured dish (right panel). The quantitative data are expressed as means ± SD. N= 3; significant differences: **, p-value* < 0.05.

**Figure Supplementary 5 (video).** Engineering 3D vascularized bone platform. Confocal immunofluorescence video capturing the endothelial vessel surrounding by HOBs: ECs (red), HOBs (green), nuclei for DAPI (blue).

**Figure Supplementary 6 (video).** Dextran assay. Representative real-time imaging of 70 kDa dextran assay in presence of osteoblast mineralization media (MM).
